# Enhanced Acetaminophen Electrochemical Sensing Based on Nitrogen-Doped Graphene

**DOI:** 10.3390/ijms232314866

**Published:** 2022-11-28

**Authors:** Lidia Magerusan, Florina Pogacean, Stela Pruneanu

**Affiliations:** National Institute for Research and Development of Isotopic and Molecular Technologies, Donat Street, No. 67-103, 400293 Cluj-Napoca, Romania

**Keywords:** graphite electrochemical exfoliation, nitrogen-doped graphenes, modified electrodes, acetaminophen electrochemical detection

## Abstract

Because of the widespread acetaminophen usage and the danger of harmful overdosing effects, developing appropriate procedures for its quantitative and qualitative assay has always been an intriguing and fascinating problem. A quick, inexpensive, and environmentally friendly approach based on direct voltage anodic graphite rod exfoliation in the presence of inorganic salt aqueous solution ((NH_4_)_2_SO_4_–0.3 M) has been established for the preparation of nitrogen-doped graphene (exf-NGr). The XRD analysis shows that the working material appears as a mixture of few (76.43%) and multi-layers (23.57%) of N-doped graphenes. From XPS, the C/O ratio was calculated to be 0.39, indicating a significant number of structural defects and the existence of multiple oxygen-containing groups at the surface of graphene sheets caused by heteroatom doping. Furthermore, the electrochemical performances of glassy carbon electrodes (GCEs) modified with exf-NGr for acetaminophen (AMP) detection and quantification have been assessed. The exf-NGr/GCE-modified electrode shows excellent reproducibility, stability, and anti-interfering characteristics with improved electrocatalytic activity over a wide detection range (0.1–100 µM), with a low limit for AMP detection (LOD = 3.03 nM). In addition, the developed sensor has been successfully applied in real sample analysis for the AMP quantification from different commercially available pharmaceutical formulations.

## 1. Introduction

The need for effective over-the-counter medicine is rising daily. Acetaminophen (N-acetyl-p-aminophenol, AMP), also known as paracetamol, an acylated aromatic amide, the major metabolite of phenacetin and acetanilide, is among the most extensively used medications worldwide, being recommended by the World Health Organization (WHO) as first-line therapy in different pain conditions [1]. Both a painkiller and a fever reducer, since its approval in 1951, acetaminophen has been available in a variety of forms, including: suppositories, injections, normal pills, chewable tablets, capsules, suspension or solution, effervescent tablets, and syrups. AMP is often employed in conjunction with opioids to alleviate severe to moderate discomfort associated with backache, toothache, sore throat, arthritis, headache, muscular pains, colds, flu, and fever [2]. At typical therapeutic concentrations, acetaminophen undergoes rapid conjugation with glutathione and is mainly metabolized in the liver via first-order kinetics, producing both cysteine and mercapturic acid conjugates [3]. However, AMP overdoes (consumption of a single dose greater than 7 g in an adult and 150 mg/kg in a child [4] can result in the build-up of toxic metabolites, which can cause severe and occasionally deadly hepatotoxicity and nephrotoxicity [5]. Furthermore, a variety of other medications have been reported to interact with acetaminophen, increasing its toxicity [6,7,8]. As a consequence, AMP has been reported as one of the most common sources of drug poisoning from either accidental or intentional overdose and is the most prevalent drug-related cause of acute liver failure [9]. Additionally, it is responsible for numerous deaths and emergency department visits worldwide [10]. Thus, in order to reduce any possible hepatotoxicity risk, healthcare practitioners are mandated to avoid prescribing and distributing medications containing more than 325 mg AMP/dose [11]. Under these circumstances, caution is necessary, and the most important factor in acetaminophen toxicity is prevention. Therefore, the development of effective, sensitive, and straightforward analytical techniques for its detection is crucial for the medical management of biological fluids (plasma, blood, and urine) as well as quality control of pharmaceutical formulations. According to a literature review, in its pure form or in combination with other medications, AMP was determined by different analytical tools, including: titrimetry [12,13], spectrofluorimetry [14,15], spectrophotometry [16,17], gas chromatography-mass spectrometry (GC-MS) [18], high-performance liquid chromatography (HPLC) [19], high-performance thin layer chromatography [20], HPLC–MS/MS [21], micellar electrokinetic capillary (MEC) [22], and flow injection chemiluminescence [23]. However, these procedures are often expensive, sophisticated, and time consuming, and they require highly qualified specialists to operate, severely restricting their wider utility. Electrochemical approaches, on the other hand, have several benefits, such as affordability, simplicity, rapidity, and user friendliness, being preferred to classical analysis methods. Over the past decades, a variety of materials have been employed as electrode modifiers in order to enhance AMP detection: bismuth oxide nanoparticles [24], molecularly imprinted polymeric micelles [25], electropolymerized-molecularly imprinted polypyrrole [26], poly(Patton and Reeder’s reagent) [27], carbon nanotubes [28]; poly (L-Leucine)-layered carbon nanotube [29], multiwalled carbon nanotube and β-cyclodextrin [30], nafion/TiO_2_-graphene [31], and graphene oxide-Y_2_O_3_ nanocomposite [32].

Among all, carbon materials with heteroatom doping have generated a great deal of attention in the creation of electrochemical sensors because of their extraordinary electrocatalytic activity [33]. Numerous efforts have been made to add heteroatoms to graphene sheets as an efficient way to modify their structure and characteristics [34]. Particularly nitrogen doping has become a popular issue as a feasible way to enhance the graphene properties since an active redox center might form when nitrogen atoms are present, causing pseudocapacitance to improve the specific capacitance of the materials [35]. Up to date, a variety of chemical routes have been developed and employed for N-doped graphene preparation, including chemical vapor deposition (CVD) [36], thermal annealing [37], pyrolysis [38], arc-discharge method [39], plasma treatment [40], hydrothermal method [41], solvothermal method [42], microwave-assisted technique [43], wet chemical reaction [44], and flame treatment [45]. However, the large-scale, economic, and easy preparation of N-doped graphene material is still a challenge.

In this study, a one-step, eco-friendly electrochemical strategy for the synthesis of nitrogen-doped graphene has been devised, and the applicability of the as-prepared material in the acetaminophen electrochemical assay is presented. Compared to other reported results on the same topic, the current study has the advantage of the easy, rapid, and low-cost preparation method of electrode material since in situ homogeneous heteroatom doping is achieved via directly adding nitrogen-containing compounds as electrolyte solution during the electrochemical exfoliation of graphite rods.

## 2. Results and Discussion

### 2.1. Morpho-Structural Material Characterization

**Figure 1:**
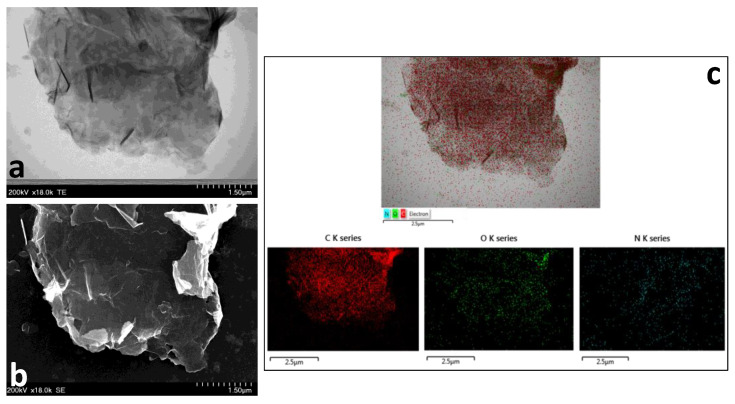
TEM/SEM micrographs corresponding to exf-NGr material (**a**,**b**); EDX mapping of C, O, and N atoms (**c**).

The N-doped graphene morphology was revealed by TEM/SEM micrographs (Figure 1a,b). The flakes generally have large sizes (in the range of micrometers) and a silk-like appearance with few wrinkles. The EDX mapping (Figure 1c) disclosed a uniform distribution of C, O, and N elements on the graphene flake surface, validating the homogeneous heteroatom doping. The dark lines visible in the TEM images account for the graphene sheets overlapping and defects present in the carbon-based hexagonal structure. Due to the heteroatom doping, the lone electrons pair of N atoms can detach the sp^2^-hybridized C atoms from their conjugated systems, and the activation of π electrons leads to charge distribution and a certain surface defect degree [46], explaining the wrinkled surface.

**Figure 2:**
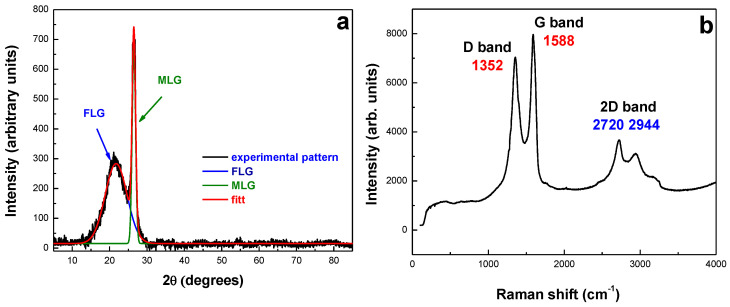
The XRD diffraction pattern (**a**) and Raman spectrum (**b**) of exf-NGr material.

XRD analysis provides valuable information regarding the exf-NGr sample composition and crystallinity (see Figure 2a). The material appears as a mixture of few-layer (FLG) and multi-layer graphene (MLG) and the most intense features from the XRD pattern, located at 21.76 and 26.45 degrees, correspond to (002) crystalline framework of FLG (76.43%) and MLG (23.57%), respectively.

The XRD experimental data enabled the estimation of the number of layers (n), the interlayer spacing (d), and the mean crystallite size (D). Using the Debye–Scherer equation [47], the D value for each peak was computed from the corresponding full width at half maximum (FWHM). Further, the Bragg equation [48] was employed to determine the d value, while the average number of layers, n, was calculated using the n = D/d relation (see Table 1).

As visible, the interlayer distance (3.37 Å) of MLG is slightly higher than that corresponding to pure graphite (3.34 Å), while for FLG, this value is even larger (4.08 Å) since the electrochemical oxidation process of graphite in the presence of ammonium sulphate increases the interplanar spacing generating the separation of graphene layers, concomitant with in situ heteroatom doping. This leads to the attachment of functional groups and defect formation. As a result, the final product is a blend of 3 and 27 layers of N-doped graphenes, with a predominant proportion of a few layers. In addition, there is a considerable difference between the crystallite sizes of FLG and MLG, from 13.28 nm to 93.06 nm.

Raman spectroscopy was further used to establish the samples’ composition and crystallinity. As depicted in Figure 2b, the two spectral features located at 1352 cm^−1^ (D) and 1588 cm^−1^ (G) are correlated to sp^3^ carbon domains and sp^2^ bonds in the graphitic network, respectively [49]. The position of these bands is in agreement with other results from the literature [50]. It is well known that the D band represents clear evidence for the presence of structural defects, and the intensity ratio of D and G bands is a quantitative measure of the disorder degree [51]. In our case, I_D_/I_G_ equals 0.88, confirming the existence of some structural instability that disrupts the translational symmetry of the hexagonal graphene structure as a result of carbon hybridization by heteroatom–carbon bond formation [52]. Furthermore, according to the empirical equation developed by Knight and White [53], there is a strong correlation between I_D_/I_G_ ratio and the in-plane crystallite size (L_a_): I_D_/I_G_ = C(λ)/L_a_, where C(λ) is a wavelength-dependent factor. For the exf-NGr sample, the L_a_ value was determined to be 18.86 nm.

**Figure 3:**
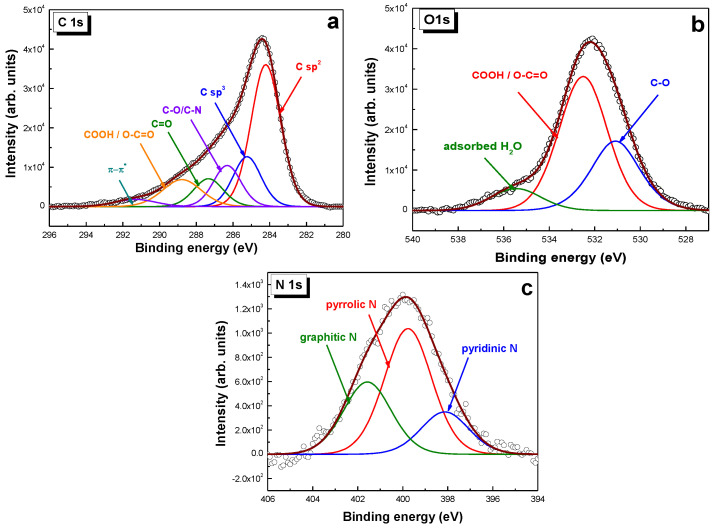
Deconvoluted high–resolution XPS spectra of C1s (**a**), O 1s (**b**), and N 1s (**c**) states corresponding to exf-NGr.

XPS analysis was employed as a valuable tool to analyse the surface composition and chemical state of the N-doped graphenes. High-resolution XPS measurements corresponding to C, O, and N states are shown in Figure 3. Six components were found by *C 1s* peak deconvolution (see Figure 3a and Table S1 from Electronic Supplementary Materials). The major contributions centred at 284.21 eV and 285.23 eV correspond to sp^2^ and sp^3^ hybridized carbon atoms, respectively, whereas the other smaller intensity peaks in the range of 286–290 eV are generated by C-O, O-C=O, C-N, C=O bonding. In the high binding energy of the spectrum, a minor contribution assigned to the π → π^*^ shake-up satellite band of graphitic carbons could be observed [54]. The sp^2^ carbon hybridization is representative of the graphene-conjugated honeycomb lattice, while the presence of sp^3^ hybridized carbons indicates the replacement of N atoms, defects in graphene, or simply the edges of the graphene nanosheets [50]. The presence of structural defects is further confirmed by the deconvolution of *O 1s* states (Figure 3b and Table S1 from Electronic Supplementary Materials) in the form of three different contributions assigned to carbon–oxygen-bonded groups (C-O, C=O/O-C=O) and adsorbed water molecules. The C/O ratio was determined to be 0.39, suggesting a high number of structural defects and, thus, the presence of numerous oxygen-containing functionalities at the surface of graphene sheets induced by heteroatom doping.

The existence of different carbon-nitrogen bonding types in the graphene lattice is confirmed by high-resolution *N1s* peak deconvolution (Figure 3c and Table S1 from Electronic Supplementary Materials). Similar to other reports on N-doped graphenes [34,55], the three distinct components centred at binding energies: 398.12 eV, 399.76 eV, and 401.57 eV are associated with pyridinic, pyrrolic, and graphitic nitrogen contribution. The main contribution accounting for 52.13% of the total nitrogen corresponds to pyrrolic, a state that, of the aforementioned three N kinds, is responsible for enhancing the electrical properties of doped graphene sheets by providing delocalized electrons [56]. The C/N ratio was found to be 17.36, confirming a small doping degree.

### 2.2. Electrochemical Analysis

#### 2.2.1. Modified Electrode Surface Area Determination

In order to evaluate the active areas of bare and modified GCE, their voltammetric behavior was tested using a standard 1 mM K_4_[Fe(CN)_6_] redox probe in 0.2 M KCl supporting electrolyte at different scan rates from 2 to 100 mV/s. The registered cyclic voltammograms show a reversible process on the modified electrode surface (see Figure S1a from Electronic Supplementary Materials), and the anodic maximum plotted against the square root of scan rate follows a linear regression equation defined as: I_p_ = 2.34∙10^−7^ + 3.5∙10^−5^ × υ^1/2^ (R^2^ = 0.998)—Figure S1b from Electronic Supplementary Materials. Further, the electrode area was estimated using the Randles–Ševcik equation [57], obtaining several modified electrodes values between 0.047 and 0.052 cm^2^, proving the immobilization of N-doped graphenes at the surface of a glassy carbon electrode and the excellent reproducibility of the electrode modification process. In all cases, the determined active area was considerably higher than that corresponding to the bare electrode (0.028 cm^2^), indicating a larger current response, which may lead to an increased sensitivity and a lower detection limit.

**Figure 4:**
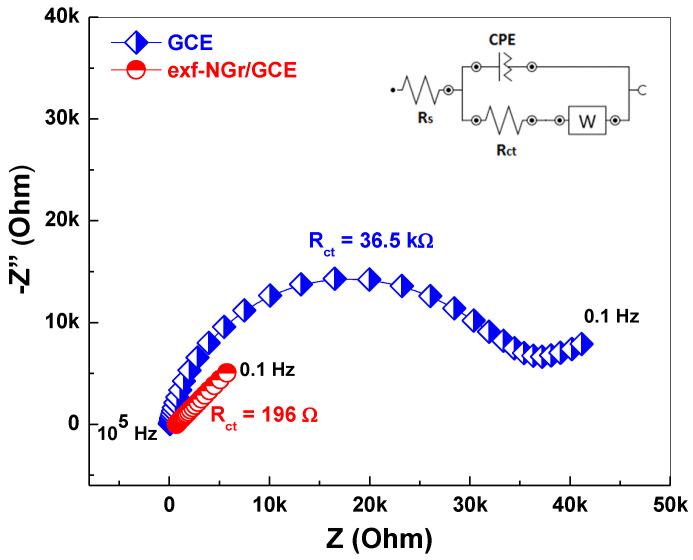
Nyquist plot of the bare GCE (blue curve) and exf-NGr/GCE (red curve)*. Inset*: The electrical equivalent circuit employed to fit the experimental results.

Using electrochemical impedance spectroscopy, the electrodes capacity for electron transfer was examined. The EIS spectra for bare and graphene-modified electrode are presented in Figure 4, along with the equivalent electrical circuit employed to fit the experimental results (inset).

The circuit contains the solution resistance (R_s_), the charge-transfer resistance (R_ct_), which characterizes the easiness of electron transfer at the electrode/solution interface, the Warburg impedance (W), which characterizes the diffusion of the ions, and, finally, a constant phase element (CPE), which replaces the double-layer capacitance, due to the high porosity of the interface. As expected, there are marked differences between the two spectra. For GCE, the R_ct_ value is very high (36.5 kΩ) in contrast with that corresponding to the exf-NGr/GCE-modified electrode (196 Ω). This indicates that the exf-NGr sample not only increases the active area of the modified electrode but also accelerates the transfer of electrons.

Based on the R_ct_ value, the apparent heterogeneous electron transfer rate constant (K_app_) for each electrode was calculated using the equation: K_app_ = RT/n^2^F^2^AR_ct_C [58], where n is the number of electrons transferred during the redox reaction; F is the Faraday constant (96,485 C/mol); R is the ideal gas constant (8.314 J/mol·K); T is the temperature (298 K); A is the active area of the electrode (cm^2^); R_ct_ is the charge-transfer resistance (Ω); and C is the analyte concentration (mol/cm^3^). For bare GCE, K_app_ was determined to be 2.58 × 10^−4^ cm/s, while after exf-NGr immobilization, it increased almost 2 orders of magnitudes (2.59 × 10^−2^ cm/s). The result further proved that exf-NGr material is an excellent candidate for developing electrochemical sensors.

#### 2.2.2. Acetaminophen Electrochemical Behavior at the Surface of exf-NGr/GCE

**Figure 5:**
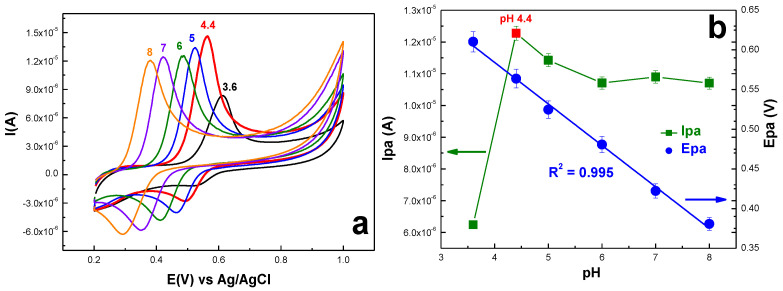
(**a**) Voltammetric response of exf-NGr/GCE surface in buffer solutions containing 100 µM AMP at different pH values (3.6, 4.4, 5.0, 6.0, 7.0, and 8.0); scan rate: 10 mV/s; (**b**) variation of the peak current with pH value (green curve) and linear dependence of peak potential vs. pH value (blue curve).

**Scheme 1:**
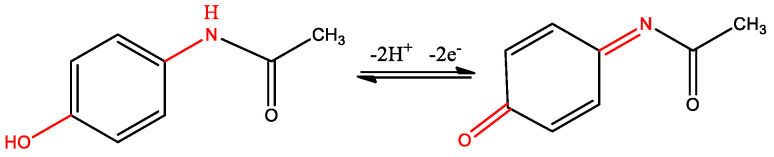
Suggested AMP electrochemical mechanism at the surface of the exf-NGr/GCE-modified electrode.

CV is a powerful method that depicts the material’s behavior in catalyzing the molecule’s redox reaction. Figure 5 shows the pH influence across a range of 3.6 up to 8 for AMP (100 µM) electrochemical behavior on the exf-NGr/GCE-modified electrode. Well-defined anodic and cathodic waves were recorded at each pH value, denoting a quasi-reversible electrochemical mechanism (Figure 5a). The results indicate that the anodic peak intensity increases up to a maximum registered at pH 4.4 and then falls off when the pH of the solution continues to rise (green curve from Figure 5b). Furthermore, the protonation process was also evident since the peak potential changed toward negative potentials as the pH increased. The slope of the anodic peak potential vs. pH plot was found to be −52 mV/pH (blue curve from Figure 5b), close to the Nernstian theoretical value of 59 mV/pH [59], indicating that proton transfer was present during the AMP electrochemical redox process. Furthermore, one can conclude that an equal number of electrons and protons are involved in the reaction [60], and a possible mechanism defined by a quasi-reversible reaction was proposed (see Scheme 1) in accordance with previously reported studies of Alam et al. [61], Hanabaratti et al. [62], and Mahmoud et al. [63].

**Figure 6:**
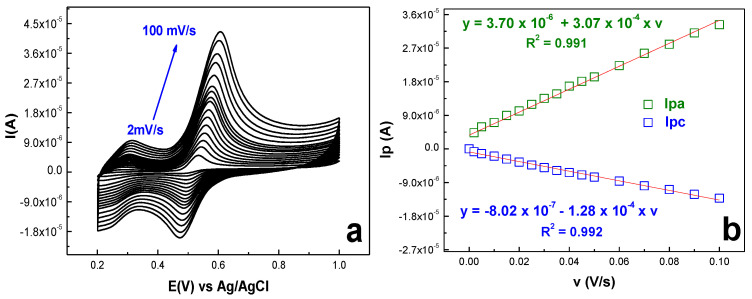
CVs recorded in 100 µM AMP (pH 4.4) at various scan rates, from 2 to 100 mV/s (**a**); variation of the anodic and cathodic peak currents with the scan rate (**b**).

In order to determine if the AMP electrochemical oxidation at the surface of the exf-NGr/GCE-modified electrode is a diffusion or adsorption-controlled process, different scan rates were employed. Thus, 100 µM AMP in pH 4.4 acetate was used as the test substance, and cyclic voltammograms were acquired, varying the scan rate between 2 and 100 mVs^−1^ (see Figure 6a). The increase in scan rates resulted in a positive shift in the oxidation peak potential and a negative shift in the reduction peak potential. Furthermore, for both oxidation and reduction peak currents, a linear dependence with the scan rate could be observed, as shown in Figure 6b.

The scan rate-dependent behavior follows a linear regression equation defined as: I_pa_ = 3.70 × 10^−6^ + 3.07 × 10^−4^∙*v* (R^2^ = 0.991) and I_pc_ = −8.02 × 10^−7^ − 1.28 × 10^−4^∙*v* (R^2^ = 0.992) for the anodic and cathodic peak, respectively, confirming that AMP electrochemical reaction at the surface of exf-NGr/GCE-modified electrode is a surface-controlled process [64]. The quasi-reversible character of the AMP reaction, in which the rate of electron transfer becomes comparable to the mass transport rate, may be used to explain the positive shifting of the oxidation peak potentials and the negative shifting of the reduction peak potentials as the scan rate increases [65].

#### 2.2.3. Calibration Curve and Limit of Detection

**Figure 7:**
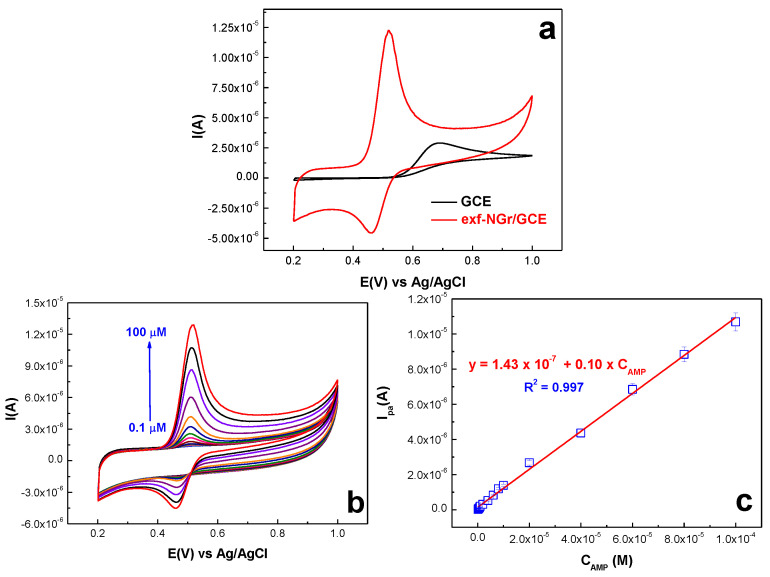
(**a**) Cyclic voltammograms of bare GCE and exf-NGr/GCE-modified electrodes recorded in acetate buffer (4.4) in the presence of 100 µM AMP, scan rate 10 mV/s (**b**) CVs recorded with exf-NGr/GCE-modified electrode in acetate buffer solution containing different AMP concentration (0.1–100 µM), scan rate 10 mV/s; (**c**) corresponding calibration curve (peak intensity vs. AMP concentration) for exf-NGr/GCE-modified electrode.

The electrochemical performances of exf-NGr/GCE-modified electrode toward AMP (100 µM) were compared to those of bare GCE electrode (see Figure 7a), indicating a considerable enhancement of the graphene-modified surface sensing abilities (five times higher). The unmodified electrode shows a broad oxidation maximum centered at +688 mV and a small reduction at +533 mV, while the modified electrode exhibits well-defined oxidation and reduction peaks at 519 and 460 mV, respectively. Thus, the peak-to-peak separation decreased from 155 mV for GCE to 59 mV in the case of exf-NGr/GCE. In addition, the sensing performances of exf-NGr/GCE were investigated over a wide AMP concentration range, 0.1–100 µM (see Figure 7b), and determining that the anodic peak current increases proportionally with AMP concentration (Figure 7c) obeying a linear regression equation defined as: I_pa_ = 1.43∙10^−7^ + 0.10 × C_AMP_ (R^2^ = 0.997). The limit of quantification (LOQ—defined as the smallest analyte amount at which measurements could be performed with reasonable accuracy and precision) was determined to be 0.1 µM while the limit of detection (LOD—defined as the minimum analyte amount that can be consistently detected but not precisely measured) [66].

**Figure 8:**
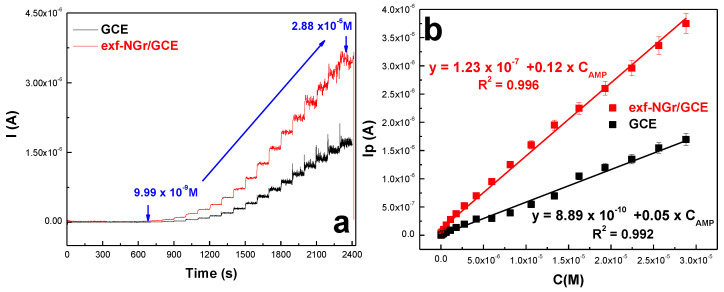
(**a**) Amperometric curves recorded with exf-NGr/GCE (red; +0.52 V applied potential) and GCE (black; +0.7 V applied potential) in pH 4.4 supporting electrolyte, after the addition of AMP from 10^−4^ M stock solution; the concentration range was from 9.99 × 10^−9^ to 2.88 × 10^−5^ M AMP; (**b**) the corresponding calibration plots for exf-NGr/GCE (red) and GCE (black), using background subtracted signals.

Better results were obtained by employing the amperometric technique, since the technique is known to be more sensitive than classical CV. The recorded amperograms can be seen in Figure 8a, while the corresponding calibration plots are presented in Figure 8b, for both electrodes. The linear range for AMP obtained with exf-NGr/GCE was very wide, from 0.009 to 28.8 µM, and the detection limit was very low, 3.03 nM. The sensitivity was 0.12 A/M, being three times higher than that of bare GC (0.05 A/M), proving the advantages of using N-doped graphene for the AMP electrochemical detection.

The electrochemical performances of developed exf-NGr/GCE-modified electrode were compared to other reports from literature. As shown in Table 2, the developed sensor is similar or outperforms prior studies in terms of linear detection range and detection limit. The enhanced functionality can be ascribed to the incorporation of nitrogen atoms in the graphene structure, which increases the number of defects generating additional active sites. Furthermore, the lone pair of electrons from the nitrogen atoms serves as carriers, raising the graphene-based material charge density, enhancing the modified electrode conductivity [67].

#### 2.2.4. Reproducibility, Accuracy, Stability, and Interference Effects

In order to assess the reproducibility of GCE surface modification, the electrochemical signals of five electrodes modified under identical circumstances were recorded and compared (see Figure S2a from Electronic Supplementary Materials; 100 µM AMP solution prepared in pH 4.4 acetate buffer). The variances in anodic peak current intensity do not surpass 5.91%, showing that the working electrode preparation is reproducible. Investigations were also conducted on the sensor’s stability. The electrode retains at least 87.72% of its initial response while subjected to 50 consecutive CV scans in acetate buffer (pH 4.4 solution) containing 100 µM AMP, at a scan rate of 10 mV/s (see Figure S2b from Electronic Supplementary Materials). In addition, the sensor was stored in ultrapure water over a long time period (more than 6 months) and its accuracy and long-time stability were evaluated recording its CV current response to 100 µM AMP from time to time. The results indicate that the modified electrode retains 94.53% of its initial response over a time period that exceeds 7 months (see Figure S2c from Electronic Supplementary Materials). These findings attest to the sensor’s excellent repeatability and stability.

The impact of different interfering species or a mixture of interfering species was studied to determine the modified electrode selectivity. First CV measurements were performed in acetate buffer solution pH 4.4 containing 100 µM AMP combined with different organic molecules (uric acid—UA, ascorbic acid—AA, citric acid—CA, glucose—GCZ, cysteine—CYS, tartaric acid—TA) at two fix concentrations of 10 µM and 100 µM. As shown in Figure S3a from Electronic Supplementary Materials, the presence of uric acid in the working solution generated the apparition of a small peak located at approximately 0.51 V, corresponding to uric acid electrochemical oxidation at the surface of N-doped graphene-modified surface. The UA peak is clearly separated from the one generated by AMP oxidation. All the other molecules do not give rise to any additional contribution in the AMP cyclic voltammograms (see Figure S3b–f). In the presence of UA, AA, CA, GCZ, and CYS (concentration 100 µM), the AMP signal is only slightly affected, while TA produces a decrease in anodic peak intensity (as visible in Figure S3f from Electronic Supplementary Materials). A slight decrease in the voltammetric signal was also recorded when the interference was set at an equal amount with the analyte of interest (100 µM) for CA, GCZ, and TA, but the modified electrode still retains over 80% of its initial response. The presence of a complex interference matrix does not seem to significantly affect the AMP electro-oxidation process, as suggested by the results obtained in Figure S3g from Electronic Supplementary Materials, indicating that the developed sensor is a suitable candidate for real sample analysis.

#### 2.2.5. Real Sample Analysis

**Figure 9:**
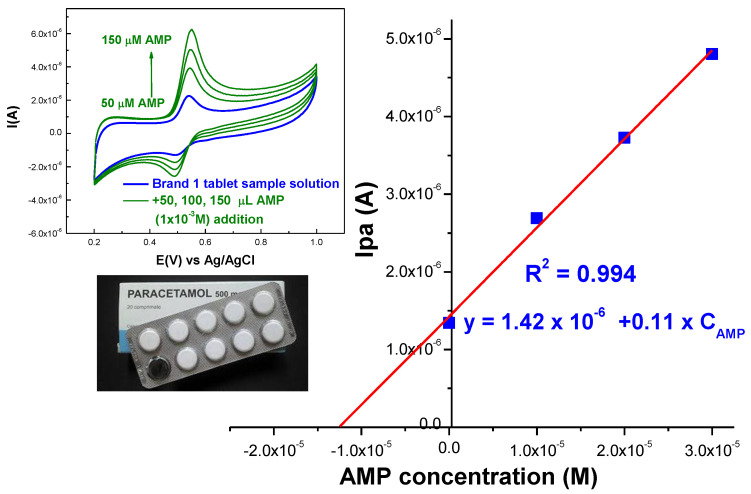
Calibration plot obtained for the standard addition method in case of paracetamol-containing solutions obtained from Brand 1 real tablet sample; *Inset*: CVs recorded after spiking AMP from the stock solution (1 mM) supporting electrolyte acetate buffer (pH 4.4) solution; scan rate 10 mV/s.

In order to test the practical ability of exf-NGr/GCE-modified electrodes, different brands of pharmaceutical tablet formulations containing acetaminophen (500 mg/tablet) were examined for the presence of labeled paracetamol content. First, the tablets were weighted, crushed, and grounded to form a homogeneous fine powder. The corresponding quantity of the powdered tablet was accurately weighted and transferred in 10 mL acetate buffer solution (pH 4.4) in order to obtain a real sample solution of 0.1 mg/mL AMP. Further, aliquots of 100 µL of the as-prepared paracetamol-containing tablet solution were transferred in acetate buffer solution up to a maximum of 5 mL (which resulted in a final concentration of 13.2 µM AMP). This was regarded as the ‘unidentified’ concentration that had to be discovered. Next, three different volumes (50, 100, and 150 µL) from AMP standard stock solution (1 mM) were spiked in three different beakers, each containing aliquots of 100 µL paracetamol-containing tablet solution 0.1 mg/mL (the total volume in each beaker was set to 5 mL) and cyclic voltammograms were recorded for each of the as-prepared solutions (see green curves from the insets of Figure 9 and Figure S4a,b from Electronic Supplementary Materials). Then, the oxidation peak current was plotted versus the added AMP concentration, and the unknown AMP concentration in the real sample solution was determined from the calibration plot (Figure 9 and Figure S4).

The findings for AMP detection in pharmaceutical drugs are shown in Table 3. The results are in suitable agreement with those obtained using UV-Vis as a complementary analytical technique (see Table 3 and Figure S5 from Electronic Supplementary Materials), demonstrating the suggested method’s accuracy and, consequently, its applicability for determining AMP in actual samples, including tablet formulation.

## 3. Materials and Methods

### 3.1. Reagents

All chemical materials were of the highest purity and used as they were, with no further purification, unless otherwise mentioned. Acetaminophen (AMP, C_8_H_9_NO_2_, ≥99.0%), graphite rods (6 mm diameter, 99.995% purity), potassium chloride (KCl, 99.98%), uric acid (UA, C_5_H4N_4_O_3_, 99%), and L-(+)-Tartaric acid (TA, C_4_H_6_O_6_, ≥99.5%) were obtained from Sigma-Aldrich Chemie GmbH (Steinheim, Germany). Sodium dihydrogen phosphate (H_2_NaO_4_P, 100%) and disodium hydrogen phosphate anhydrous (HNa_2_O_4_P, 99.7%) were acquired from VWR BDH Chemicals (Leuven, Belgium). Sodium acetate anhydrous (CH_3_COONa, ≥99.0%), sodium thiosulfate pentahydrate (Na_2_S_2_O_3_·5H_2_O), and ammonium sulfate ((NH_4_)_2_SO_4_, ≥99.0%) were purchased from ChimReactiv SRL (Bucuresti, Romania). Potassium hexacyanoferrate(III) (K_4_[Fe(CN)_6_, ≥99%), L(+)-ascorbic acid (AA, C_6_H_8_O_6_, 99.7%) were acquired from Merck (Darmstadt, Germany). Citric acid (CA, C_6_H_8_O_7_, 99+%) and L-cysteine (CYS, C_3_H_7_NO_2_S, 98+%) were acquired from Alpha Aesar GmbH & Co KG (Karlsruhe, Germany) aqueous solutions were freshly prepared using ultrapure water (18.2 MΩ, Milli-Q water purification system), protected from light by refrigeration at 4 °C when not in use.

### 3.2. Synthesis of Nitrogen-Doped Exfoliated Graphenes

A rapid, low-cost, and environmentally friendly protocol has been developed for nitrogen-doped graphene preparation based on direct voltage anodic graphite exfoliation in the presence of inorganic salt. Shortly, an electrochemical exfoliation cell was set in a 150 mL Berzelius beaker. Next, two graphite rods (6 mm diameter), fixed with the aid of a Teflon holder at a distance of 2 cm, were immersed in the beaker containing 100 mL ammonium sulphate solution (0.3 M). Then, a low bias of 6 V was applied for about 4 h from a power supply (Mesit DC; Mařatice, Slovakia). Under these mild conditions, shortly after the experiment started, the anode surface became rough, and exfoliated graphene flakes appeared in the solution. At the end of the 4 h, a very concentrated dark-brown solution was obtained, and it was left to rest overnight. The next day, the two phases separated, the black colour product settled at the bottom of the electrochemical cell, and so it was filtered and rigors washed with a high quantity of ultrapure water to remove the ammonium sulphate salt. The nitrogen-doped graphene sample was finally obtained via the freeze-drying method, and it was further denoted exf-NGr.

### 3.3. Instruments

*Transmission electron microscopy (TEM), scanning electron microscopy (SEM), and scanning transmission electron microscopy (STEM) with energy-dispersive X-ray (EDX) spectroscopy technique for the elemental mapping of C, O, and N atoms distribution* were performed using a SU-8230 STEM system (Hitachi, Japan). CuK_α1_ radiation (λ = 1.5406 Å), filtered with a Ge (111) monochromator, from a Brucker D8 Advance Diffractometer, over the 2θ range of 3–85° was employed to collect the X-ray diffraction (XRD) patterns. Raman measurements were performed at room temperature using a JASCO-NRS 3300 Spectrophotometer arranged in backscattering geometry, equipped with a CCD detector (−68 °C), Ar-ion laser (514 nm) with the power at the sample surface of 1.3 mW, an incident laser beam (of about 1 μm^2^) focused with an Olympus Microscope and a 100× objective, using a 600 L/mm grid, spectral resolution of 14.6 cm^−1^. X-ray Photoelectron Spectroscopy (XPS) measurements were performed under irradiation with an Al_Kα_ X-ray source (1486.6 eV) operated at 200 W, using a SPECS spectrometer, equipped with a dual-anode X-ray source Al/Mg, a PHOIBOS 150 2DCCD hemispherical energy analyzer and a multi-channeltron detector. The XPS survey spectrum was recorded at 30 eV pass energy, 0.5 eV/step. The high-resolution spectra for individual elements were recorded by accumulating 15–20 scans at 30 eV pass energy and 0.1 eV/step. The surface cleaning was ensured through argon ion bombardment at 500 V for 5 min. The sample did not show electrostatic charging; thus, the binding energies are presented without any correction. The raw data were used with no preliminary smoothing. Data analysis and experimental curve fitting of the C 1s, O 1s, and N 1s spectra were performed using Casa XPS software—version 2.3.16 (Casa Software Ltd., Wilmslow, Cheshire, UK), with a Gaussian-Lorentzian product function and a non-linear Shirley background correction. UV-Vis spectra were acquired in the 200–800 nm range using a SPECORD 250 PLUS spectrophotometer (Analytik Jena GmbH, Jena, Germany). A Milli-Q water purification system (Biobase Biotech(jinan) Co., Ltd, Jinan, China) was used to prepare ultrapure water (18.2 MΩ). In order to determine the pH levels of the solutions, an Apera PC9500 research-grade benchtop multiparameter was employed, and the dispersions were assured with an ultrasonic bath Elmasonic P 120 H (1000 W).

### 3.4. Electrochemical Experiments

#### 3.4.1. Preparation of Nitrogen-Doped Graphene-Modified Electrodes (exf-NGr/GCE)

The modified electrode was prepared via the drop-casting method, depositing successive layers on the surface of a clean, glassy carbon electrode from a homogeneous solution prepared by blending 1.5 mg exf-NGr material with 2 mL DMF. The optimization studies revealed that a total volume of 8 µL exf-NGr/DMF solution was necessary to obtain the best electrochemical performances compared to the bare GCE. Smaller graphene colloidal solution volumes were not enough to obtain a suitable electrochemical signal, while for higher volumes, the electronic transport during the redox process is hampered by the thick coating, which is reflected in the decrease in the peak current. The obtained electrode was denoted as exf-NGr/GCE, and it was further employed for the AMP electrochemical detection.

#### 3.4.2. Electrochemical Setup

Cyclic voltammetry (CV), chronoamperometry, and electrochemical impedance spectroscopy (EIS) measurements were performed at room temperature for the AMP electrochemical assay at the surface of the exf-NGr/GCE-modified electrode. The experiments were carried out with a 302 N potentiostat/galvanostat (Metrohm-Autolab B.V., Kanaalweg, Utrecht, The Netherlands) controlled by Nova 1.11 version, using a three-electrode setup, with Ag/AgCl (KCl at 3 M) as a reference, platinum wire as a counter electrode, and the modified exf-NGr/GCE as a working electrode. The potential range was set between (0.2–1) V, and CVs were recorded at scan rates ranging between 2 and 100 mV/s. Besides being used for the electrochemical behavior of bare and all modified electrodes, the CV technique was also applied to investigate the active surface area of the electrodes, the scan rate and pH effect in the study of AMP electrochemical oxidation, interference studies, and real sample analysis. The amperometric analysis was performed at +0.51 V for exf-NGr/GCE and +0.68 V for GCE. The EIS spectra were recorded within 0.1–10^5^ Hz at an applied potential of +0.263 V and +0.241 V for the bare and exf-NGr/GCE, respectively (5 mV excitation signal). It is worth mentioning that after each measurement, the working electrode was electrochemically cleaned by successive CV scans (>50 cycles), within 0.2….1 V potential range, in acetate buffer solution (pH 4.4) at a scan rate of 50 mV/s. Furthermore, between measurements, the modified electrode was permanently kept in ultrapure water at room temperature to ensure a complete desorption process.

#### 3.4.3. Preparation of Working Solutions

The supporting electrolyte acetate buffer solution (pH 3.6, 4.4, and 5) was prepared by mixing specific amounts of sodium acetate and acetic acid solution in double distilled water while supporting electrolyte of phosphate buffer solution (PBS, pH 6, 7, and 8) was obtained by mixing equimolar (0.1 M) sodium dihydrogen phosphate and disodium hydrogen phosphate anhydrous in distilled water, followed by adjusting the pH value with additional drops of HCl (0.1 M) and NaOH (0.1 M). A stock AMP solution (1 mM) was prepared by dissolving 3.78 mg acetaminophen in 25 mL acetate buffer (pH 4.4), and working solutions of different concentrations (in the range of 0.1–100 µM) were obtained by dilution. For real sample preparation, three paracetamol tablets (different brands, all labeled 500 mg paracetamol/tablet) were weighed and further ground to powder. A total of 1 mg of the homogenized powdered tablet from each brand was transferred in 10 mL acetate buffer solution (pH 4.4). Further aliquots of 100 µL from the obtained real sample solutions were diluted in acetate buffer (pH 4.4) up to a maximum volume of 5 mL; then CV and UV-Vis measurements were performed. For the standard addition, four different beakers containing aliquots of 100 µL from the prepared tablet sample solution and the corresponding volume of acetate buffer (up to a maximum volume of 5 mL) were spiked with three different volumes (50, 100, and 150 µL) from a stock AMP solution (1 mM); the corresponding voltammetric signals were recorded, and the acetaminophen content in the real sample solution was calculated. All experiments were performed in triplicate.

## 4. Conclusions

An economic and easy method to obtain a sensor for sensitive and selective acetaminophen detection and quantification was developed from glassy carbon electrode surface modification with N-doped graphene. The exf-NGr/GCE sensor shows improved electrocatalytic activity toward the acetaminophen oxidation process, with a linear dependence of the oxidative peak current on a wide concentration range (0.1–100) µM and a limit of detection of 30.3 nM when applying the CV method and (0.009–28.8) µM with LOD = 3.03 nM while using the amperometric technique. The method’s usefulness for determining paracetamol in different brands of tablet formulations was proven by its satisfactory recovery result, above 93.13% of the labeled content. Therefore, the developed sensor may be successfully applied in low-cost sensing systems for AMP assay.

## Figures and Tables

**Table 1:** The XRD parameters of exf-NGr material.

	2θ	D	d	n	%
**FLG**	21.76	13.28	4.08	3.25	76.43
**MLG**	26.45	93.06	3.37	27.61	23.57

**Table 2:** Electrochemical performances comparison toward AMP detection for different types of modified electrodes.

Modified Electrode	Method	Linear Range (µM)	LOD (µM)	Reference
**MWCNTs-GNS/GCE** MWCNTs-GNS—multiwalled carbon nanotubes-graphene nanosheet nanocomposite GCE—glassy carbon electrode	DPV	90–112	9	[68]
**MWCNT-β-CD/GCE** MWCNT-β-CD—multiwalled carbon nanotubes-β-cyclodextrin GCE—glassy carbon electrode	DPV	0.05–1 1–300	0.0115	[30]
**MoS_2_@NHCSs^x^/GCE** MoS_2_@NHCSs—nitrogen-doped hollow carbon spheres wrapped with MoS_2_ nanosheets GCE—glassy carbon electrode	DPV	0.05–20	0.02	[69]
**PCA@Zn/Ni-ZIF-8–800/GCE** PCA@Zn/Ni-ZIF-8–800—poly caffeic acid (PCA)-Metal-organic frameworks Zn/Ni-ZIF-8–800 GCE—glassy carbon electrode	DPV	0.08–1000	0.029	[70]
**Cr-SBC/GCE** Cr-SBC—poly(chromium Schiff base complex) GCE—glassy carbon electrode	DPV	0.008–0.125	0.0056	[71]
**PEDOT/GCE** PEDOT—poly(3,4-ethylenedioxythiophene) GCE—glassy carbon electrode	DPV	1–100	0.4	[72]
**GO-Y/CPE** GO-Y CPE—carbon paste electrode	DPV	7–400	1.45	[32]
**CNTs/SPCE** CNTs—carbon nanotubes SPCE—screen-printed electrode	CV	2.5–1000	0.1	[73]
**BDDE** BDDE—boron-doped diamond electrode	CV	10–100	0.97	[74]
**MWCNT-BPVCM/GCE** MWCNT-BPVCM—branched amphiphilic photo-sensitive and electroactive polymer-multiwalled carbon nanotubes hybrid GCE—glassy carbon electrode	CV	5–1000	3.5	[75]
**Graphene/GCE** GCE—glassy carbon electrode	CV	0.1–20	0.032	[76]
**GI/GCE** GI—graphene ink GCE—glassy carbon electrode	CV	10–500	2.7	[77]
**PDDA-GR/GCE** PDDA-GR—poly(diallyl- dimethylammonium chloride) functionalized graphene GCE—glassy carbon electrode	CV	20–200	0.221	[78]
**GO/GCE** GO—graphene oxide GCE—glassy carbon electrode	SWV	0.05–1	0.049	[79]
EFTAG/CPE EFTAG—ethyl 2-(4-ferrocenyl-[1,2,3]triazol-1-yl)-graphene CPE—carbon paste electrode	SWV	1–150	0.5	[80]
**BDDE** BDDE—boron-doped diamond electrode	SWV	2.99–283	0.768	[81]
**RGO-CB-CTS/GCE** RGO-CB-CTS—reduced graphene oxide-carbon black-chitosan GCE—glassy carbon electrode	SWV	2.8–19	0.053	[82]
**FeS/rGO/GCE** FeS/rGO—FeS anchored reduced graphene oxide nanosheets GCE—glassy carbon electrode	Amperometry	2–8	0.1	[83]
**Ni-Al-HCF LDH/GCE** Ni-Al-HCF LDH—Ni-Al-hexacyanoferrate (III)-layered double hydroxides GCE—glassy carbon electrode	Amperometry	3–1500	0.8	[84]
**Protonated g-C_3_N_4_/CTS-GCE** Protonated g-C_3_N_4_ /CTS graphitic carbon nitride nanosheets GCE—glassy carbon electrode	Amperometry	1.7–2020	0.15	[85]
**RGO-gold dendrite/GCE** RGO-gold dendrite—reduced graphene oxide-supported gold dendrite GCE—glassy carbon electrode	Amperometry	0.07–3000	0.005	[86]
**GrNF/GCE** GrNF—graphene nanoflakes GCE—glassy carbon electrode	Amperometry	0.001–150	0.004	[87]
**exf-NGr/GCE**	Amperometry	0.009–28.8	0.003	current work
**exf-NGr/GCE**	CV	0.1–100	0.03	current work

CV—cyclic voltammetry, DPV—differential pulse voltammetry, SWV—square wave voltammetry.

**Table 3:** Summary of the detected acetaminophen contents of different commercially available tablet samples.

Tablet (500 mg AMP/Tablet) *Analyzed Solution* *Concentration 13.2 µM*	Detected AMP Content (µM)		* Recovery (%)
	CV	UV-Vis	
***Brand 1***	12.9	14.1	97.72
***Brand 2***	12.6	13.7	95.45
***Brand 3***	11.18	13.3	84.69

* according to CV.

## Data Availability

The data presented in this study are available upon request from the corresponding author.

## Supplementary Materials

The following supporting information can be downloaded at: https://www.mdpi.com/article/10.3390/ijms232314866/s1.

## References

[1] Ennis Z.N., Dideriksen D., Vaegter H.B., Handberg G., Pottegard A. (2016). Acetaminophen for Chronic Pain: A Systematic Review on Efficacy. Basic Clin. Pharmacol. Toxicol..

[2] Bannwarth B., Pehourcq F. (2003). Pharmacologic basis for using paracetamol: Pharmacokinetic and pharmacodynamic issues. Drugs.

[3] Mazaleuskaya L.L., Sangkuhl K., Thorn C.F., FitzGerald G.A., Altman R.B., Klein T.E. (2015). PharmGKB summary: Pathways of acetaminophen metabolism at the therapeutic versus toxic doses. Pharmacogenet. Genom..

[4] Hodgman M.J., Garrard A.R. (2012). A review of acetaminophen poisoning. Crit. Care Clin..

[5] Chiew A.L., Buckley N.A. (2021). Acetaminophen Poisoning. Crit. Care Clin..

[6] Kostrubsky S.E., Sinclair J.F., Strom S.C., Wood S., Urda E., Stolz D.B., Wen Y.H., Kulkarni S., Mutlib A. (2005). Phenobarbital and phenytoin increased acetaminophen hepatotoxicity due to inhibition of UDP-glucuronosyltransferases in cultured human hepatocytes. Toxicol. Sci..

[7] Perucca E., Richens A. (1979). Paracetamol disposition in normal subjects and in patients treated with antiepileptic drugs. Br. J. Clin. Pharmacol..

[8] Minton N.A., Henry J.A., Frankel R.J. (1988). Fatal paracetamol poisoning in an epileptic. Hum. Toxicol..

[9] Wang X., Wu Q., Liu A., Anadón A., Rodríguez J.L., Martínez-Larrañaga M.R., Yuan Z., Martínez M.A. (2017). Paracetamol: Overdose-induced oxidative stress toxicity, metabolism, and protective effects of various compounds in vivo and in vitro. Drug Metab. Rev..

[10] Lee W.M. (2017). Acetaminophen (APAP) hepatotoxicity-Isn’t it time for APAP to go away?. J. Hepatol..

[11] Mitka M. (2014). FDA asks physicians to stop prescribing high-dose acetaminophen products. JAMA.

[12] Kumar K.G., Letha R. (1997). Determination of paracetamol in pure form and in dosage forms using N,N-dibromo dimethylhydantoin. J. Pharm. Biomed. Anal..

[13] Srivastava M.K., Ahmad S., Singh D., Shukla I.C. (1985). Titrimetric determination of dipyrone and paracetamol with potassium hexacyanoferrate(III) in an acidic medium. Analyst.

[14] Llorent-Martínez E., Šatínský D., Solich P., Ortega-Barrales P., Molina-Díaz A. (2007). Fluorimetric SIA optosensing in pharmaceutical analysis: Determination of paracetamol. J. Pharm. Biomed. Anal..

[15] Vilchez J.L., Blanc R., Avidad R., Navalón A. (1995). Spectrofluorimetric determination of paracetamol in pharmaceuticals and biological fluids. J. Pharm. Biomed. Anal..

[16] Rele R.V. (2016). Simultaneous UV-spectrophotometric estimation of paracetamol and guaiphenesin by first order derivative method in combined dosage form. Res. J. Pharm. Technol..

[17] Glavanović S., Glavanović M., Tomišić V. (2016). Simultaneous quantitative determination of paracetamol and tramadol in tablet formulation using UV spectrophotometry and chemometric methods. Spectrochim. Acta A Mol. Biomol. Spectrosc..

[18] Belal T., Awad T., Clark R. (2009). Determination of paracetamol and tramadol hydrochloride in pharmaceutical mixture using HPLC and GC-MS. J. Chromatogr. Sci..

[19] Acheampong A., Gyasi W.O., Darko G., Apau J., Addai-Arhin S. (2016). Validated RP-HPLC method for simultaneous determination and quantification of chlorpheniramine maleate, paracetamol and caffeine in tablet formulation. SpringerPlus.

[20] Farid N.F., Abdelaleem E.A. (2016). HPTLC method for the determination of paracetamol, pseudoephedrine and loratidine in tablets and human plasma. J. Chromatogr. Sci..

[21] Li H., Zhang C., Wang J., Jiang Y., Fawcett J.P., Gu J. (2010). Simultaneous quantitation of paracetamol, caffeine, pseudoephedrine, chlorpheniramine and cloperastine in human plasma by liquid chromatography-tandem mass spectrometry. J. Pharm. Biomed. Anal..

[22] Emre D., Özaltın N. (2007). Simultaneous determination of paracetamol, caffeine and propyphenazone in ternary mixtures by micellar electrokinetic capillary chromatography. J. Chromatogr. B Biomed. Appl..

[23] Ruengsitagoon W., Liawruangrath S., Townshend A. (2006). Flow injection chemiluminescence determination of paracetamol. Talanta.

[24] Zidan M., Tee T.W., Abdullah A.H., Zainal Z., Kheng G.J. (2011). Electrochemical oxidation of paracetamol mediated by nanoparticles bismuth oxide modified glassy carbon electrode. Int. J. Electrochem. Sci..

[25] Luo J., Fan C., Wang X., Liu R., Liu X. (2013). A novel electrochemical sensor for paracetamol based on molecularly imprinted polymeric micelles. Sens. Actuators B Chem..

[26] Özcan L., Şahin Y. (2007). Determination of paracetamol based on electropolymerized-molecularly imprinted polypyrrole modified pencil graphite electrode. Sens. Actuators B Chem..

[27] Thomas T., Mascarenhas R.J., Cotta F., Guha K.S., Swamy B.E., Martis P., Mekhalif Z. (2013). Poly(Patton and Reeder’s reagent) modified carbon paste electrode for the sensitive detection of acetaminophen in biological fluid and pharmaceutical formulations. Colloids Surf. B Biointerfaces.

[28] Charithra M.M., Manjunatha J.G. (2020). Enhanced voltammetric detection of Paracetamol by using carbon nanotube modified electrode as an electrochemical sensor. J. Electrochem. Sci. Eng..

[29] Pushpanjali P.A., Manjunatha J.G., Hareesha N., D’Souza E.S., Charithra M.M., Prinith N.S. (2021). Voltammetric analysis of antihistamine drug cetirizine and paracetamol at poly (L-Leucine) layered carbon nanotube paste electrode. Surface. Inter..

[30] Alam A.U., Qin Y., Howlader M.M.R., Hu N.-X., Deen M.J. (2018). Electrochemical sensing of acetaminophen using multi-walled carbon nanotube and β-cyclodextrin. Sens. Actuators B Chem..

[31] Fan Y., Liu J.H., Lu H.T., Zhang Q. (2011). Electrochemical behaviour and voltammetric determination of paracetamol on Nafion/TiO_2_-graphene modified glassy carbon electrode. Colloids Surf. B Biointerfaces.

[32] Martínez-Sánchez C., Montiel-González F., Rodríguez-González V. (2019). Electrochemical sensing of acetaminophen using a practical carbon paste electrode modified with a graphene oxide-Y_2_O_3_ nanocomposite. J. Taiwan Inst. Chem. Eng..

[33] Qu L., Liu Y., Baek J.B., Dai L. (2010). Nitrogen-doped graphene as efficient metal-free electrocatalyst for oxygen reduction in fuel cells. ACS Nano.

[34] Xu H., Ma L., Jin Z. (2018). Nitrogen-doped graphene: Synthesis, characterizations and energy applications. J. Energy Chem..

[35] Yang Y., Shi W., Zhang R., Luan C., Zeng Q., Wang C., Li S., Huang Z., Liao H., Ji X. (2016). Electrochemical exfoliation of graphite into nitrogen-doped graphene in glycine solution and its energy storage properties. Electrochim. Acta.

[36] Wei D.H., Liu Y.Q., Wang Y., Zhang H.L., Huang L.P., Yu G. (2009). Synthesis of N-Doped Graphene by Chemical Vapor Deposition and Its Electrical Properties. Nano Lett..

[37] Li M., Wu Z.S., Ren W.C., Cheng H.M., Tang N.J., Wu W.B., Zhong W., Du Y.W. (2012). The doping of reduced graphene oxide with nitrogen and its effect on the quenching of the material’s photoluminescence. Carbon.

[38] Lin Z.Y., Waller G.H., Liu Y., Liu M.L., Wong C.P. (2013). 3D Nitrogen-doped graphene prepared by pyrolysis of graphene oxide with polypyrrole for electrocatalysis of oxygen reduction reaction. Nano Energy.

[39] Guan L., Cui L., Lin K., Wang Y.Y., Wang X.T., Jin F.M., He F., Chen X.P., Cui S. (2011). Preparation of few-layer nitrogen-doped graphene nanosheets by DC arc discharge under nitrogen atmosphere of high temperature. Appl. Phys. A.

[40] Soin N., Roy S.S., Roy S., Hazra K.S., Misra D.S., Lim T.H., Hetherington C.J., Malaughlin J.A. (2011). Enhanced and stable field emission from in situ nitrogen-doped few-layered graphene nanoflakes. J. Phys. Chem. C.

[41] Sun L., Wang L., Tian C.G., Tan T.X., Xie Y., Shi K.Y., Li M.T., Fu H.G. (2012). Nitrogen-doped graphene with high nitrogen level via a one-step hydrothermal reaction of graphene oxide with urea for superior capacitive energy storage. RSC Adv..

[42] Choi C.H., Park S.H., Chung M.W., Woo S.I. (2013). Easy and controlled synthesis of nitrogen-doped carbon. Carbon.

[43] Xin Y.C., Liu J.G., Jie X., Liu W.M., Liu F.Q., Yin Y., Gu J., Zou Z.G. (2012). Preparation and electrochemical characterization of nitrogen doped graphene by microwave as supporting materials for fuel cell catalysts. Electrochim. Acta.

[44] Zhang Y.J., Fugane K., Mori T., Niu L., Ye J.H. (2012). Wet chemical synthesis of nitrogen-doped graphene towards oxygen reduction electrocatalysts without high-temperature pyrolysis. J. Mater. Chem..

[45] Zhang Y.P., Cao B., Zhang B., Qi X., Pan C.X. (2012). The production of nitrogen-doped graphene from mixed amine plus ethanol flames. Thin Solid Films.

[46] Zhang L., Xia Z. (2011). Mechanisms of Oxygen Reduction Reaction on Nitrogen-Doped Graphene for Fuel Cells. J. Phys. Chem. C.

[47] Warren B.E. (1990). X-ray Diffraction.

[48] Pope C.G. (1997). X-Ray Diffraction and the Bragg Equation. J. Chem. Educ..

[49] Botas C., Álvarez P., Blanco C., Santamaría R., Granda M., Gutiérrez M.D., Rodríguez-Reinoso F., Menéndez R. (2013). Critical temperatures in the synthesis of graphene-like materials by thermal exfoliation—Reduction of graphite oxide. Carbon.

[50] Wang X., Sun G., Routh P., Kim D.-H., Huang W., Chen P. (2014). Heteroatom-doped graphene materials: Syntheses, properties and applications. Chem. Soc. Rev..

[51] Malard L.M., Pimenta M.A., Dresselhaus G., Dresselhaus M.S. (2009). Raman spectroscopy in graphene. Phys. Rep..

[52] Ferrari A.C., Meyer J.C., Scardaci V., Casiraghi C., Lazzeri M., Mauri F., Piscanec S., Jiang D., Novoselov K.S., Roth S. (2006). Raman spectrum of graphene and graphene layers. Phys. Rev. Lett..

[53] Knight D.S., White W.B. (1989). Characterization of diamond films by Raman spectroscopy. J. Mater. Res..

[54] Magerusan L., Pogacean F., Coros M., Socaci C., Pruneanu S., Leostean C., Pana I.O. (2018). Green methodology for the preparation of chitosan/graphene nanomaterial through electrochemical exfoliation and its applicability in Sunset Yellow detection. Electrochim. Acta.

[55] Magerusan L., Socaci C., Coros M., Pogacean F., Rosu M.-C., Gergely S., Pruneanu S., Leustean C., Pana I.O. (2017). Electrochemical Platform based on Nitrogen-doped Graphene / Chitosan Nanocomposite for Selective Pb2+ Detection. Nanotechnology.

[56] Lee D.H., Lee W.J., Kim S.O., Kim Y.H. (2011). Theory, Synthesis, and Oxygen Reduction Catalysis of Fe-Porphyrin-Like Carbon Nanotube. Phys. Rev. Lett..

[57] Brett C.M.A., Oliveira-Brett A.M. (1993). Electrochemistry, Principles, Methods and Applications.

[58] Nkosi D., Pillay J., Ozoemena K.I., Nouneh K., Oyama M. (2010). Heterogeneous electron transfer kinetics and electrocatalytic behaviour of mixed self-assembled ferrocenes and SWCNT layers. Phys. Chem. Chem. Phys..

[59] Zoski C.G. (2007). Handbook of Electrochemistry.

[60] Su W.Y., Wang S.M., Cheng S.H. (2011). Electrochemically pretreated screen-printed carbon electrodes for the simultaneous determination of aminophenol isomers. J. Electroanal. Chem..

[61] Alam A.U., Qin Y., Catalano M., Wang L., Kim M.J., Howlader M.M.R., Hu N.-X., Deen M.J. (2018). Tailoring MWCNTs and β-Cyclodextrin for Sensitive Detection of Acetaminophen and Estrogen. ACS Appl. Mater. Interfaces.

[62] Hanabaratti R.M., Tuwar S.M., Nandibewoor S.T., Gowda J.I. (2020). Fabrication and characterization of zinc oxide nanoparticles modified glassy carbon electrode for sensitive determination of paracetamol. Chem. Data Collect..

[63] Mahmoud B.G., Khairy M., Rashwan F.A., Banks C.E. (2017). Simultaneous Voltammetric Determination of Acetaminophen and Isoniazid (Hepatotoxicity-Related Drugs) Utilizing Bismuth Oxide Nanorod Modified Screen-Printed Electrochemical Sensing Platforms. Anal. Chem..

[64] Zhu W., Huang H., Gao X., Ma H. (2014). Electrochemical Behavior and Voltammetric Determination of Acetaminophen Based on Glassy Carbon Electrodes Modified with poly(4-Aminobenzoic Acid)/electrochemically Reduced Graphene Oxide Composite Films. Mater. Sci. Eng. C.

[65] Brownson D.A.C., Banks C.E. (2014). Interpreting Electrochemistry. The Handbook of Graphene Electrochemistry.

[66] Guideline ICH (2005). Validation of analytical procedures: Text and methodology. Q2 (R1).

[67] Liang W., Liu L., Li Y., Ren H., Zhu T., Xu Y., Ye B.-C. (2019). Nitrogen-rich porous carbon modified electrochemical sensor for the detection of acetaminophen. J. Electroanal. Chem..

[68] Tahereh M.A., Gholizadeh M. (2013). Simultaneous voltammetric determination of tyrosine and paracetamol using a carbon nanotube-graphene nanosheet nanocomposite modified electrode in human blood serum and pharmaceuticals. Colloids Surf. B Biointerfaces.

[69] Zhang D., Qian J., Yi Y., Kingsford O.J., Zhu G. (2019). Nitrogen-doped hollow carbon nanospheres wrapped with MoS_2_ nanosheets for simultaneous electrochemical determination of acetaminophen and 4-aminophenol. J. Electroanal. Chem..

[70] Zhang W., Zong L., Liu S., Pei S., Zhang Y., Ding X., Jiang B., Zhang Y. (2019). An electrochemical sensor based on electro-polymerization of caffeic acid and Zn/Ni-ZIF-8–800 on glassy carbon electrode for the sensitive detection of acetaminophen. Biosens. Bioelectron..

[71] Kumar S.P., Giribabu K., Manigandan R., Munusamy S., Muthamizh S., Padmanaban A., Dhanasekaran T., Suresh R., Narayanan V. (2016). Simultaneous determination of paracetamol and 4-aminophenol based on poly(chromium Schiff base complex) modified electrode at nanomolar levels. Electrochim. Acta.

[72] Mehretie S., Admassie S., Hunde T., Tessema M., Solomon T. (2011). Simultaneous determination of N-acetyl-p-aminophenol and p-aminophenol with poly(3,4-ethylenedioxythiophene) modified glassy carbon electrode. Talanta.

[73] Fanjul-Bolado P., Lamas-Ardisana P.J., Hernández-Santos D., Costa-García A. (2009). Electrochemical study and flow injection analysis of paracetamol in pharmaceutical formulations based on screen-printed electrodes and carbon nanotubes. Anal. Chim. Acta.

[74] Cofan C., Radovan C. (2008). Simultaneous chronoamperometric sensing of ascorbic acid and acetaminophen at a boron-doped diamond electrode. Sensors.

[75] Liu R., Zeng X., Liu J., Luo J., Zheng Y., Liu X. (2016). A glassy carbon electrode modified with an amphiphilic, electroactive and photosensitive polymer and with multi-walled carbon nanotubes for simultaneous determination of dopamine and paracetamol. Microchim. Acta.

[76] Kang X., Wang J., Wu H., Liu J., Aksay I.A., Lin Y. (2010). A graphene-based electrochemical sensor for sensitive detection of paracetamol. Talanta.

[77] Fu L., Xie K., Zheng Y., Zhang L., Su W. (2018). Graphene Ink Film Based Electrochemical Detector for Paracetamol Analysis. Electronics.

[78] Okoth O.K., Yan K., Liu L., Zhang J. (2016). Simultaneous electrochemical determination of paracetamol and diclofenac based on poly(diallyldimethylammonium chloride) functionalized graphene. Electroanalysis.

[79] Zidan M., Zawawi R.M., Erhayem M., Salhin A. (2014). Electrochemical Detection of Paracetamol Using Graphene Oxide-Modified Glassy Carbon Electrode. Int. J. Electrochem. Sci..

[80] Movlaee K., Beitollahi H., Reza Ganjali M., Norouzi P. (2017). Electrochemical platform for simultaneous determination of levodopa, acetaminophen and tyrosine using a graphene and ferrocene modified carbon paste electrode. Microchim. Acta.

[81] Eisele A.P.P., Valezi C.F., Sartori E.R. (2017). Exploiting the high oxidation potential of carisoprodol on a boron-doped diamond electrode: An improved method for its simultaneous determination with acetaminophen and caffeine. Analyst.

[82] Baccarin M., Santos F.A., Vicentini F.C., Zucolotto V., Janegitz B.C., Fatibello-Filho O. (2017). Electrochemical sensor based on reduced graphene oxide/carbon black/chitosan composite for the simultaneous determination of dopamine and paracetamol concentrations in urine samples. J. Electroanal. Chem..

[83] Liu X., Shangguan E., Li J., Ning S., Guo L., Li Q. (2017). A novel electrochemical sensor based on FeS anchored reduced graphene oxide nanosheets for simultaneous determination of dopamine and acetaminophen. Mater. Sci. Eng. C.

[84] Asadpour-Zeynali K., Amini R. (2017). Nanostructured hexacyanoferrate intercalated Ni/Al layered double hydroxide modified electrode as a sensitive electrochemical sensor for paracetamol determination. Electroanalysis.

[85] Liu L., Lv H., Wang C., Ao Z., Wang G. (2016). Fabrication of the protonated graphitic carbon nitride nanosheets as enhanced electrochemical sensing platforms for hydrogen peroxide and paracetamol detection. Electrochim. Acta.

[86] Yu S., Li H., Li G., Niu L., Liu W., Di X. (2018). Reduced graphene oxide-supported gold dendrite for electrochemical sensing of acetaminophen. Talanta.

[87] Meenakshi S., Pandian K., Gopinath S.C.B. (2020). Quantitative simultaneous determination of pentoxifylline and paracetamol in drug and biological samples at graphene nanoflakes modified electrode. J. Taiwan Inst. Chem. Eng..

