# Supplementary material for: Enhanced Acetaminophen Electrochemical Sensing Based on Nitrogen-Doped Graphene
Source: Int J Mol Sci. 2022 Nov 28;23(23):14866. doi: 10.3390/ijms232314866 (PMC9737486; doi:10.3390/ijms232314866)
Supplement: Supplementary file 1 [file ijms-23-14866-s001.zip › ijms-2069406-supplementary.pdf]

Electronic Supplementary Materials

Enhanced Acetaminophen Electrochemical Sensing Based on Nitrogen-Doped Graphene

Lidia Magerusan *, Florina Pogacean * and Stela Pruneanu

National Institute for Research and Development of Isotopic and Molecular Technologies, Donat Street, No. 67-103, 400293 Cluj-Napoca, Romania
* Correspondence: lidia.magerusan@itim-cj.ro (L.M.); florina.pogacean@itim-cj.ro (F.P.)

Table S1. Deconvolution results corresponding to high resolution *C1s*, *O1s* and *N1s* XPS spectra of exf-NGr material

**C 1s**			**O 1s**			**N 1s**		
position	***assignment***	***%***	***position***	***assignment***	***%***	***position***	***assignment***	***%***
**284.21**	C sp^2	47.13	531.09	C-O	31.25	398.12	pyridinic N	17.72
**285.23**	C sp^3	15.74	532.50	O-C=O/COOH	58.75	399.76	pyrrolic N	52.13
**286.31**	C-O/C-N	12.76	535.45	Adsorbed H_2O	10.00	401.57	graphitic N	30.15
**287.33**	C=O	9.27						
**288.77**	O-C=O/COOH	11.82						
**291.30**	π-π^*	3.28						

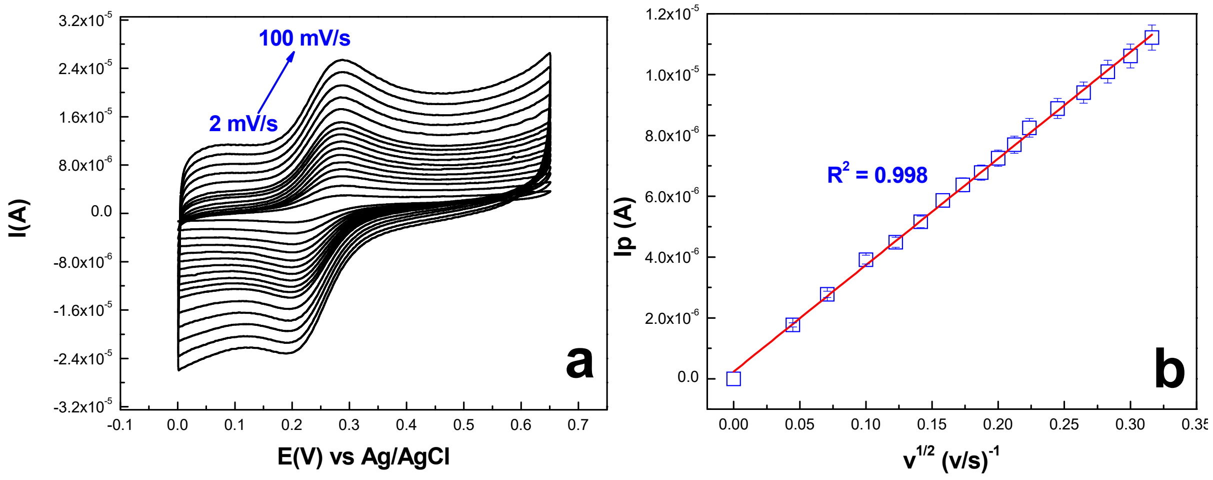

Figure S1. (a) Cyclic voltammetric response of exf-NGr/GCE surface in the presence of 1mM $K_4[Fe(CN)_6]$ at various scan rates (from 2 to 100 mV/s; 0.2 M KCl supporting electrolyte); (b) Linear plot of anodic peak current (I_p) *vs* $\upsilon^{1/2}$

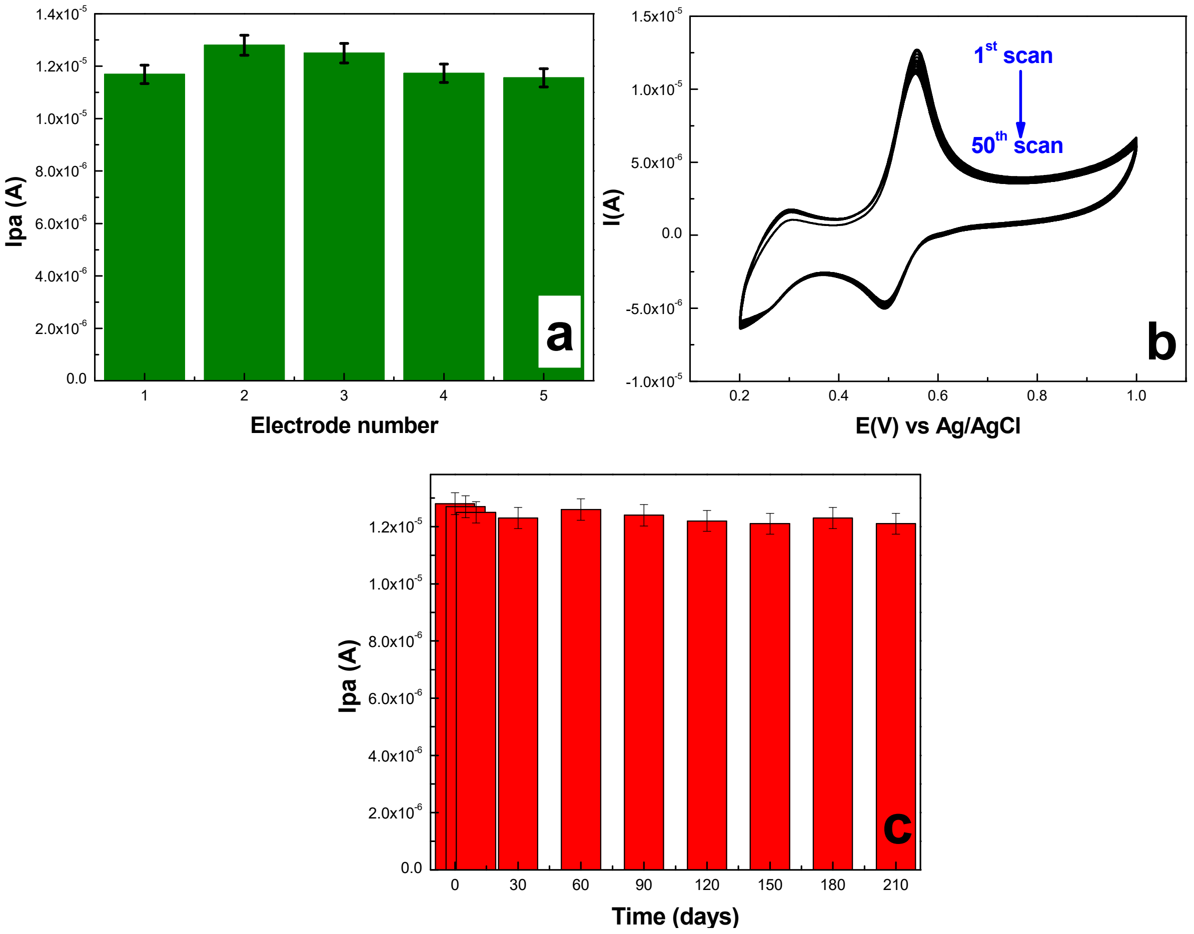

Figure S2. (a) Anodic peak current for five modified electrodes obtained from CV measurements *versus* 100 µM AMP in acetate buffer pH 4.4 solution, scan rate 10 mV/s; (c) 50 consecutive measurements in 100 µM AMP solution (pH 4.4 acetate buffer), recorded with exf-NGr/GCE (scan rate 10 mV/s); (d) CV response of exf-NGr/GCE electrode in 100 µM AMP solution (pH 4.4 acetate buffer), obtained in replicate measurements over 210 days; scan rate 10 mV/s

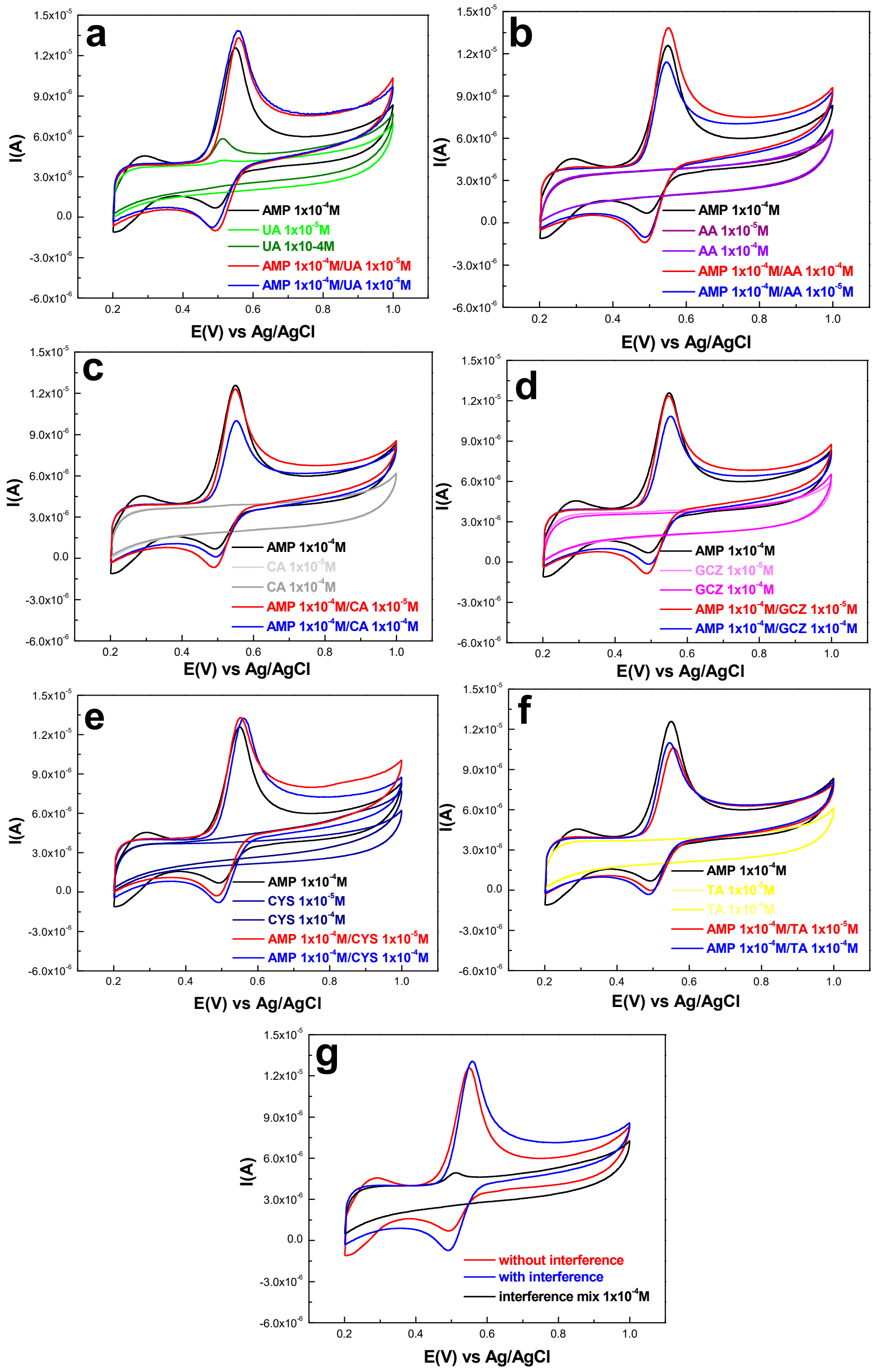

Figure S3. CV response of exf-NGr/GCE modified electrode towards 100 μM AMP in the presence of different interfering molecules - set at two fix concentration 10 μM and 100 μM): (a) uric acid (UA); (b) ascorbic acid (AA); (c) citric acid (CA); (d) glucose (GCZ); (e) cysteine (CYS); (f) tartaric acid (TA); (g) complex interfering matrix containing UA, AA, CA, GCZ, CYS and TA (100 μM); supporting electrolyte acetate buffer (pH 4.4) solution; scan rate 10 mV/s

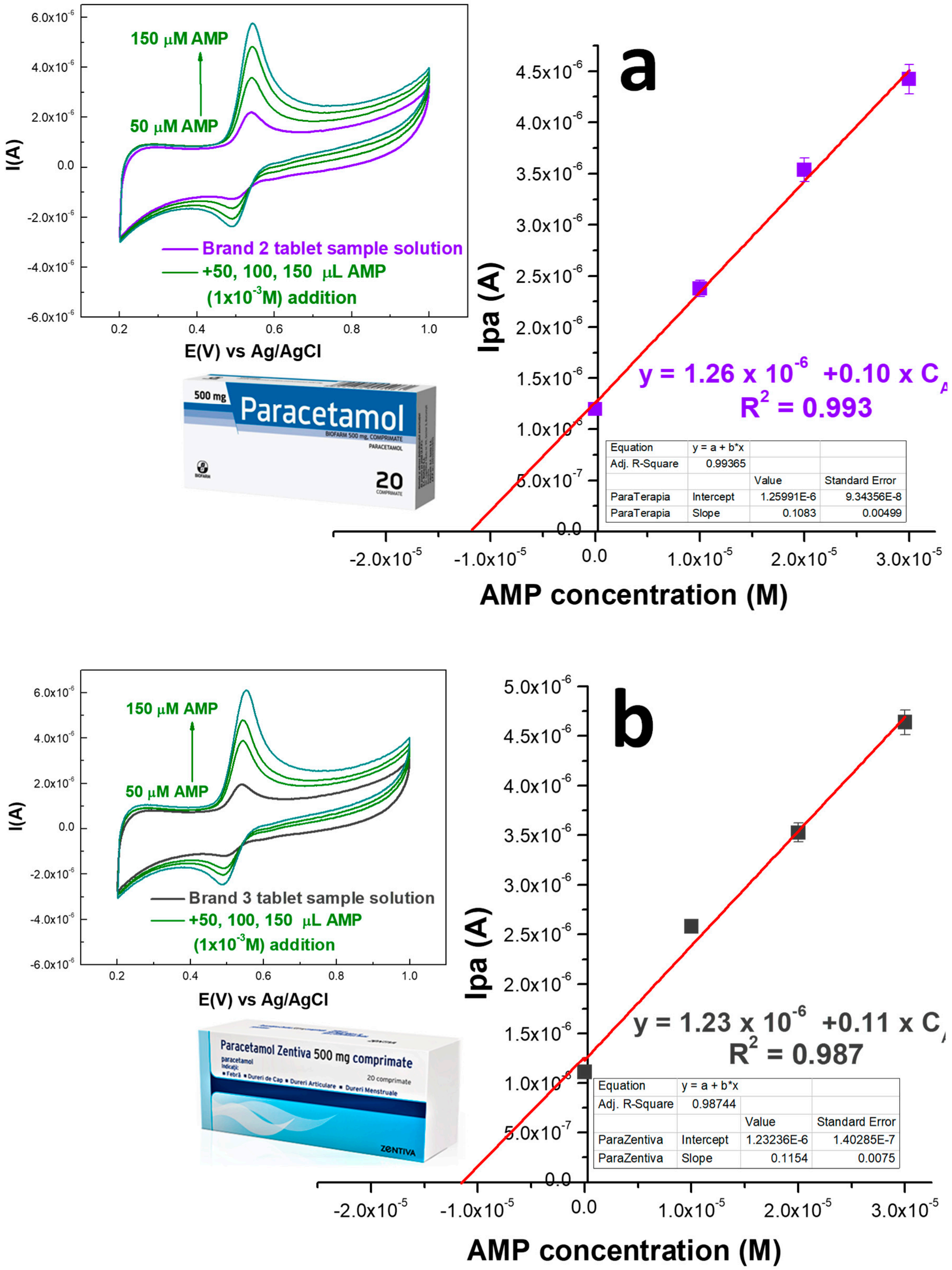

Figure S4. Calibration plot obtained for the standard addition method in case of paracetamol containing solutions obtained from Brand 2 and Brand 3 real tablet samples;

Inset: CVs recorded after spiking AMP from the stock solution (1 mM) - supporting electrolyte acetate buffer (pH 4.4) solution; scan rate 10 mV/s.

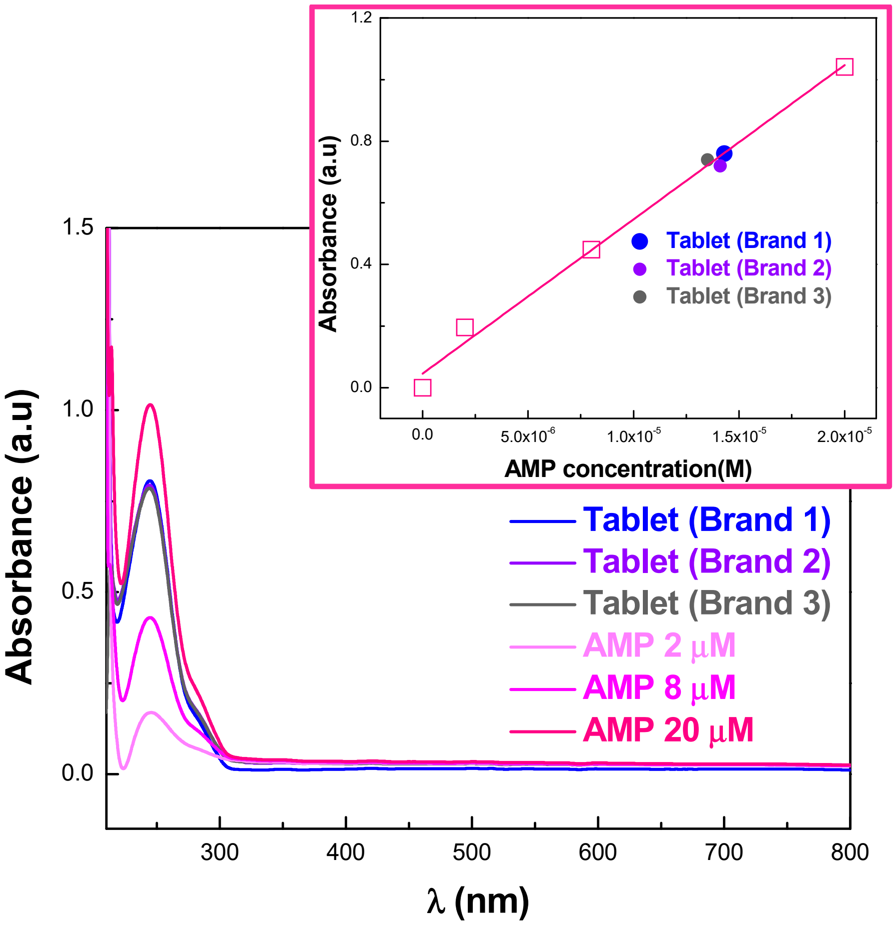

Figure S5. UV-Vis absorption spectra obtained for several solutions of known AMP concentration (2 μM; 8 μM; 20 μM) and for paracetamol containing solutions obtained from Brand 2 and Brand 3 real tablet samples (AMP concentration 13.2 μM);

Inset: The corresponding calibration curve
